# The characteristics and patterns of utilization of healthcare services among Omanis with substance use disorders attending therapy for cessation

**DOI:** 10.1371/journal.pone.0210532

**Published:** 2019-01-31

**Authors:** Nabila Al Wahaibi, Anwaar Al Lawati, Falaah Al Ruqeishy, Abdulla Al Khatri, Yahya Al-Farsi, Tahira M. A. Juma, Fatma Al Hinai, Nasser Al-Sibani, Sangeetha Mahadevan, Samir Al-Adawi

**Affiliations:** 1 Wadi Kabir Health Centre, Directorate General of Health Services, Muscat Governorate, Ministry of Health, Muscat, Oman; 2 Ruwi Health Centre, Directorate General of Health Services, Muscat Governorate, Ministry of Health, Muscat, Oman; 3 Muscat Health Centre, Directorate General of Health Services, Muscat Governorate, Ministry of Health, Muscat, Oman; 4 Al Masarrah Hospital, Ministry of Health, Muscat, Oman; 5 Department of Family Medicine and Public Health, Sultan Qaboos University, College of Medicine and Health Sciences, Muscat, Oman; 6 Directorate of Health Services, Muttrah, Muscat Governorate, Ministry of Health, Muscat, Oman; 7 Department of Behavioral Medicine, College of Medicine and Health Sciences, Sultan Qaboos University, Muscat, Oman; Geneva University Hospitals, SWITZERLAND

## Abstract

**Background:**

It is indicated that Oman is witnessing an increase in issues pertinent to alcohol and psychoactive substance use.

**Aim:**

The aim of this study was to identify the characteristics of Omanis with substance use disorder attending a specialized hospital in Oman and the pattern of their utilization of healthcare services. A related aim was to ascertain the age group most vulnerable to alcohol and substance use in Oman.

**Method:**

A cross-sectional study was conducted in a tertiary care center specialized for treatment of those engaging in substance use in Oman. The participants in the study were selected from a convenience sample among patients seeking consultation at the center for alcohol and substance use. A six-part questionnaire was designed to obtain information regarding socio-demographic background, clinical history, healthcare utilization and perceived hurdles to access. Chi-square analyses were used to evaluate the significance of differences among categorical data. Logistic regression modelling was used to obtain measures of association after adjusting for confounding factors.

**Results:**

Among the patients (n = 293) seeking cessation therapy, 99% were male and less than 30 years of age. Peer influences on the initiation of substance use were significant. Most patients had a history of polysubstance use, including intravenous substance use. Cannabis and alcohol were the first substances consumed by most patients and Hepatitis C and psychiatric disorders were found to be the most common co-morbidities. The participants that reported use of cannabis and benzodiazepines were more likely to perceive “improvement” upon receiving treatment.

**Conclusion:**

This study indicated that males below 30 years of age with a history of polysubstance use were likely to attend a hospital specialized in treating substance use disorder in Oman. This study identified information regarding socio-demographic background, risk factors and perceived hurdles to healthcare that could serve as groundwork for further studies conducted on newly emerging issues of substance use in Oman.

## Introduction

Historically, indigenous populations of the Arabian Gulf have not shown signs of non-medical use of substances [1]. Islam, having been practiced in its conservative and puritanical form in the Arabia Gulf, may have been the cause for the observation of such a trend [2]. However, there have been exceptions to this finding: countries like Yemen and Saudi Arabia have had their own long-standing tradition of chewing leaves of *Catha edulis* or *khat*, a substance promoting substance use disorder [3, 4]. Since the 17^th^ century, smoking tobacco and its variations (waterpipe/*Shisha*) have also started becoming a part of Arab tradition [5]. In the recent decades, increasing affluence has transformed this region into a hub for international trade and resulted in rapid modernization and acculturation. With the rise in demand from a growing expatriate population and the expanding tourism industry, the consumption of alcohol has also increased [6]. In their study, Al Marri and Oei [1] have indicated that the use of other psychoactive substances in this region has been on the rise as well. Much of the current population in Oman is young [7]. Studies all over the world have shown youth to be at a higher risk for non-medical use of substances compared to the rest of the population [8, 9,10]. There is an abundance of literature suggesting that youth choosing to experiment with psychoactive substances earlier are likely to face more difficulties in engaging with their lives in a meaningful manner [11, 12,13]. Thus, identifying early characteristics in the youth of Oman, that make them vulnerable to substance use, is important.

In addition to demographic change and its aftermath, Oman’s literacy and education levels have dramatically grown over the decades. Increased employment opportunities have sparked relentless urban drift [14, 15] thereby weakening traditional family ties [16]. Similar social changes, speculated elsewhere, have been shown to be associated with the spread of substance use [6, 17]. Available evidence has indicated an increase in the number of reported cases of illicit substance use [18] and psychoactive substance overdose [19] in Oman. A recent report in the ***World Health Organization’s ATLAS of Substance Use Disorders*** [20] has suggested that the prevalence of disorders related to substance use in Oman stands at 0.37% and 0.02% for alcohol and psychoactive substances, respectively. Jaffer et al’s [21] survey of risky behavior among a nationally representative sample of Omani school children (n = 3345) indicated that approximately 15% of the children had consumed tobacco or its rejuvenated forms and 4% had consumed alcohol. Another study among students in universities, colleges and other institutions indicated that 2.2% of students had previously consumed alcohol, 2.7% had experimented with psychoactive substances and 2% were sniffing inhalants [22]. The aforementioned community-based studies, however, were unclear about information regarding participants’ patterns of seeking healthcare.

Patterns of healthcare utilization among different populations have been well-studied [23] and there is growing evidence that seeking healthcare is often influenced by sociocultural factors [24]. The latter is in consonance with the fact that diseases, illnesses and disabilities are experienced within a sociocultural context that shapes and directs help-seeking behavior [25]. Cross-cultural variation in the patterns of substance use suggests that there is merit to exploring the sociocultural factors that influence healthcare-seeking behaviour in individual societies. Such studies are of particular importance in countries such as Oman where the dearth of such research is coupled with a unique religious, cultural, and geopolitical history. Most Omanis are Ibadhi, the third sect of Islam [6]. Historically, Ibadism has been documented as being intolerant towards the non-medical use of psychoactive substances [6], bringing it into theological conflict with other schools of Islam from the medieval era [26]. In addition to this, Oman’s isolation and economic underdevelopment till the early 1970s, caused it to be called ‘the Tibet of Arabia’ [27]. Little is known about the extent to which Oman’s rapid transition from isolation to urbanization and acculturation has affected its sociocultural fabric—and *inter alia* re-shaped healthcare seeking behavior among the population. This provides Oman with the added advantage of providing the perfect research ground for this emerging field of study.

Previously, various models of substance use disorder, such as the moral model, the cultural model, the habit model as well as the disease/genetic model, have been postulated [6]. In Oman, the emphasis has been on viewing alcohol and psychoactive substance use as part of the biomedical model [28]. A person with substance use disorder is thus perceived as being unwell and therefore deserving of medical treatment and rehabilitation [6, 29, 30]. A tertiary care unit has been established to meet the demands of this population, [31]. This unit has clinics specializing in the treatment of substance use disorders, i.e. providing various treatment options relevant to harm reduction and abstinence models. However, to date, there have not been any studies examining the characteristics and patterns of health care utilization among Omanis attending a hospital specializing in alcohol and substance disorders, a paucity that the current study attempts to address.

The aim of the current study was to identify socio-demographic data, factors influencing initiation into alcohol and substance use, types of substances used, co-morbid conditions, healthcare utilization, and finally, the hurdles to seeking and undergoing therapy and rehabilitation at this specialized center. Such an undertaking has the potential to promote evidence-based services for individuals with substance use disorders. Since the population of Oman has a prominent ‘youth bulge’, the related aim of the current study was to explore whether age could be a determinant of the characteristics of those who use psychoactive substances and patterns of healthcare utilization in Oman.

## Methods

This cross-sectional, descriptive study was carried out at Al Masarrah Hospital (formerly Ibn Sina Hospital), a tertiary psychiatric hospital in Oman. It is the only psychiatric hospital in Oman that has a substance use unit, which was established in 2003 [32]. This unit consists of an emergency facility, an outpatient clinic and an inpatient clinic with a capacity of 44 beds (22 for those in therapy for cessation (or reduction) of psychoactive substance use and 22 for rehabilitation). Our study was conducted across a year, from June 2013 to June 2014.

Participants were selected by convenience sampling from among Omanis aged 15 years and above and attending the outpatient non-medical substance use clinic during a one-year study period. Potential candidates were approached. Only those who fit the inclusion criteria (Omani national, cognitively intact to participate in this study, and admitted with a diagnosis of substance use disorder as defined by the *Diagnostic and Statistical Manual of Mental Disorders* DSM-IV-TR [33]) were invited by staff nurses. Urine tests were obtained from consenting patients to categorize the type of substance used. Psychiatric diagnosis was classified according to the DSM-IV-TR [33].

Our research assistants trained the nurses to ensure quality assurance. The purpose and methodology of the study were explained to the participants in detail. All potential subjects received an information sheet that covered all aspects of the study. For patients below the age of 18 (minors) invitation letters were sent to their accompanying parents/guardians describing the details of the study who were then asked to sign consent forms if they agreed to the participation of their wards. In addition to parental consent, the minors themselves were also required to provide written consent before taking part in the study.

A structured questionnaire was used to collect data during personal interviews conducted by qualified nurses and health educators. A total of 310 individuals were approached, and 298 (96.1%) consented to participate in the study. Five questionnaires that were returned with incomplete responses were excluded. Finally, a total of 293 patients were considered for analysis, resulting in a 94.5% response rate.

Prior to selecting patients to participate in this study, a questionnaire was developed based on the available literature on the Knowledge, Attitude, Practice (KAP) paradigm for substance use [34–37], with items culturally adapted to the Omani context. First, an English language version of the questionnaire was developed to accurately elicit KAP of substance use. It included both close- and open-ended questions. Close-ended questions (in a checklist format), were designed to investigate patients’ knowledge regarding substance use, while open-ended questions explored the reasons for their answers.

The questionnaire was composed of three main parts: socio-demographic data, characteristics of the patient’s alcohol and substance use, and utilization of healthcare services. The first part was designed to collect data such as age, gender, marital status, residential region, and educational level. The second part contained questions regarding the history and nature of the patient’s substance use. This included questions pertaining to the age of onset, mode of psychoactive substance intake, types of substances used, co-morbidities, and historical attributes of psychoactive substance use. The third part consisted of open-ended questions which helped investigate the participant’s utilization of healthcare services. For example, the patients who were undergoing treatment were asked about the frequency of visits to healthcare service providers, emergency visits, admissions to hospital, and hurdles to seeking healthcare services related to substance use. The English questionnaire was piloted on 25 randomly-selected patients, fluent in English, from among the target population. Following this, the questionnaire was translated into Arabic [38].

The questionnaire was then was piloted further on 25 randomly selected patients. Before filling it out, the participants were required to provide us with informed consent. The questionnaire was then subjected to evaluation by experts in psychometrics. The content and construct validity and inter-rater reliability were reported to be high (kappa = 0.86). The patients that were included in piloting samples were all included in the overall analysis of the study.

Previous studies in Oman have shown that the individuals belonging to younger age-groups, i.e. aged < 30 years, are more susceptible to risky behavior such as non-medical substance use [39]. This trend has also been observed in the United Arab Emirates (UAE), a neighboring country of Oman [40]. Thus, the patients in this study were divided into two groups, namely, *Younger* (<30 years), and *Older* (>30 years). For analysis, the dichotomous division was expected to simplify the presentation of data and facilitate conducting binary categorical data analysis [41]. But this method has its own drawbacks that will be reported later as part of the limitations of the study.

To evaluate the statistical significance of differences among proportions of categorical data, Chi-square analyses were conducted. The non-parametric Fisher’s exact test (two-tailed) replaced the Chi-square test in cases of small sample size, where the expected frequency was less than 5 in any of the cells in 2 x 2 tables. The odds ratios (OR) and 95% confidence intervals (CI) obtained from logistic regression models were taken as the measures of predictors of perceived improvement upon receiving healthcare. The variables were entered in a forward stepwise manner and Directed Acyclic Graphs (DAGs) were used to determine the confounding covariates to be included in the regression analysis. All statistical analyses were conducted using Statistical Package for Social Sciences (SPSS 20.0).

## Ethical approval

All procedures performed in this study were in accordance with the local Institutional Review Board (IRB). The study was approved by the Directorate General of Planning and Studies, Ministry of Health, Sultanate of Oman (No. 552/2016).

## Results

### Socio-demographic features of those engaging in substance use

Table 1 shows the socio-demographic characteristics of the patients who were in treatment stratified by age. Overall, the mean age (± standard deviation, SD) of the participants was 31.9 ± 8.3. Out of the 293 patients in the study, 126 (43%) were younger than 30 years of age (the Younger group), and 167 (57%) were 30 years or older (the Older group). The mean age of the Younger group was 24.4 ± 3.0, while that of the Older group was 37.4 ± 6.3. In total, there were only 3 females undergoing treatment for substance use disorder. A majority of the patients were unmarried and unemployed males residing in the governorate of Muscat and had obtained pre-secondary education.

**Table 1:** Socio-demographic features among attendees seeking consultation for alcohol and substance dependence at Al Masarra Hospital, Oman, from June 2013 to June 2014.

Characteristic	Total (N = 293)	Younger (< 30 years) (N = 126)	Older (≥ 30 years) (N = 167)	P value
	n (%)	n (%)	n (%)	
**Gender**				0.13
Male	290 (99)	126 (100)	164 (98)	
Female	3 (1)	0	3 (2)	
**Region**				0.16
Muscat	230 (79)	103 (82)	127 (76)	
Batinah North	33 (11)	15 (12)	18 (11)	
Others	30 (10)	8 (6)	22 (13)	
**Marital status**				0.02
Single	172 (58)	99 (79)	73 (44)	
Married	102 (35)	25 (20)	77 (46)	
Divorced/Widow	19 (7)	2 (1)	17 (10)	
**Education level**				0.01
Pre-secondary school	153 (52)	53 (42)	100 (60)	
Secondary school	117 (40)	64 (51)	53 (32)	
Higher education	23 (8)	9 (7)	14 (8)	
**Employment status**				0.17
Unemployed	153 (52)	72 (58)	81 (49)	
Employed	136 (47)	52 (41)	84 (50)	
Student	4 (1)	2 (1)	2 (1)	

### Characteristics related to psychoactive substance use

Table 2 provides the characteristics of alcohol and substance use among patients that consented to participate in this study. Reported age of onset for substance use was between 15 and 19 years. Motives stated for substance use were substance use by peers, peer influence, problem-relief, and curiosity. About two-thirds of the sample population identified injection as their mode of substance use. The majority (82%) reported polysubstance use. Only 10% reported single substance use, while the alcohol-only category constituted 8%. The most commonly used substance was opioid (78%) followed by cannabis (71%), alcohol (63%), and heroin (58%). These were followed by benzodiazepines, cocaine, and inhalants. Infectious diseases were common co-morbidities.

**Table 2:** Characteristics among attendees seeking consultation for alcohol and substance use at Al Masarra Hospital, Oman, from June 2013 to June 2014.

Characteristic	Total (N = 293)	Younger (< 30 y) (N = 126)	Older (≥ 30 y) (N = 167)	P value
	n (%)	n (%)	n (%)	
**Age of onset**				0.001
10 to 14	48 (16)	22 (17)	26 (16)	
15 to 19	144 (49)	73 (58)	71 (43)	
20 to 24	56 (19)	23 (18)	33 (20)	
25 or more	44 (15)	8 (6)	36 (22)	
**Motive of substance use**				
Curiosity	87 (30)	34 (27)	53 (32)	0.37
Have a good time	74 (25)	34 (27)	40 (24)	0.55
Friends are doing it	102 (35)	46 (37)	66 (40)	0.59
Ease another problem	97 (33)	34 (27)	63 (38)	0.05
Others	29 (10)	8 (6)	21 (13)	0.07
**Mode of substance intake**				0.02
Injection	195 (67)	93 (74)	102 (61)	
Non-injection	98 (33)	33 (26)	65 (39)	
**Substance type**				0.001
Alcohol only	22 (8)	0	22 (13)	
Single substance use	30 (10)	8 (6)	22 (13)	
Polysubtance use	241 (82)	116 (92)	125 (75)	
**Types of substances used**				
Opioids	227 (77)	105 (83)	122 (73)	0.04
Cannabis	207 (71)	101 (80)	106 (63)	0.002
Alcohol	185 (63)	73 (58)	112 (67)	0.11
Heroin	170 (58)	81 (64)	89 (53)	0.05
Benzodiazepines	148 (51)	65 (52)	83 (50)	0.75
Cocaine	61 (21)	21 (17)	40 (24)	0.13
Inhalants	22 (8)	9 (7)	13 (8)	0.84
**Co-morbidities**				
Hepatitis C virus	121 (41)	57 (45)	64 (38)	0.23
Depression	102 (35)	44 (35)	54 (32)	0.97
Suicidal attempt	52 (18)	27 (21)	25 (15)	0.15
Urethral/ vaginal discharge	23 (8)	4 (3)	19 (11)	0.26
HIV/AIDS	12 (4)	1 (1)	11 (7)	0.01
Hepatitis B virus	9 (3)	2 (2)	7 (4)	0.20
Tuberculosis (TB)	1 (0.3)	1 (0.8)	0 (0)	0.25
**Historical attributes of use**				
Ease to get alcohol	225 (77)	92 (73)	133 (80)	0.18
Ease to get psychoactive substances	182 (62)	81 (64)	101 (60)	0.51
Childhood traumatic experience	104 (35)	48 (38)	56 (34)	0.42
Family history of substance use	103 (35)	44 (35)	59 (35)	0.94
Neglect	56 (19)	23 (18)	33 (20)	0.74
Physical abuse	25 (9)	17 (13)	8 (5)	0.008
Sexual abuse	4 (1)	1 (0.8)	3 (2)	0.46

More than 40% of the participants reported being infected by hepatitis C virus (HCV), while HIV constituted about 4%, closely followed by hepatitis B (HBV) (3%), and TB (0.1%). Depression and suicidal attempts were reported to happen among 35% and 18% of the patients respectively, after alcohol and substance use. Compared to the Older group, the Younger group had a significantly earlier age of onset for substance use. The motives of substance use were generally similar among the two groups, except that a significantly higher proportion among the Older group reported that they used psychoactive substances to ease their ‘problems’. Intake through injection was significantly higher among the Younger group (74% vs. 61%; *p*: 0.02). More people reported use of polysubstance among the Younger group (92% vs. 75%). There were also higher proportions of use of opioids, cannabis, heroin, and benzodiazepines reported within the Younger group. The differences were statistically significant in the case of opioids (*p*: 0.04), cannabis (*p*: 0.002), and heroin (*p*: 0.05). While higher rates of infectious diseases, depression, and suicidal attempts were seen among the Younger group, these differences were not statistically significant (*p*: >0.05).

Table 2 also identifies the historical attributes of the participants’ alcohol and substance use. Majority of them reported that they found it easy to obtain alcohol and psychoactive substances. More than a third reported a family history of substance use disorder (35%) and more than a third reported undergoing a traumatic experience during childhood (35%). 44 out of the 293 participants (15.0%) reported both a positive family history of substance use disorder and a traumatic experience during childhood. Additionally, they reported a history of neglect (19%), physical abuse (9%), and sexual abuse (1%) during childhood as well. The historical attributes were similar among the Younger and Older groups except for physical abuse, which was significantly higher for the Younger group (14% vs. 5%; *p*: 0.008).

### Patterns of utilization of healthcare services

Table 3 presents results regarding the utilization of healthcare services. Overall, participants reported a total of 697 visits to the facility. The data showed us that 39% were OPD and emergency visits. Admissions constituted 22% of the total visits. The majority (78%) reported visiting OPD regularly, and 58% reported obtaining services in the emergency department. Those who were previously admitted constituted about 55%. The most common reason identified for recent emergency visits was relapse, followed by suicide attempts, overdose and lack of medication. A commonly reported reason for non-emergency admissions was also relapse, in addition to depressive symptoms, overdose, and suicide attempts.

**Table 3:** Patterns of utilization of healthcare services among attendees seeking consultation for alcohol and substance use at Al Masarra Hospital, Oman, from June 2013 to June 2014.

Characteristic	Total (N = 293)	Younger (< 30 y) (N = 126)	Older (≥ 30 yrs) (N = 167)	P value
	N (%)	N (%)	N (%)	
**Visits to healthcare services**				0.08
Total	697 (100)	247 (100)	450 (100)	
Outpatient	272 (39)	112 (45)	160 (35)	
Emergency	272 (39)	103 (42)	169 (38)	
Admission	153 (22)	32 (13)	121 (27)	
**OPD Attendance**				0.56
Regular	228 (78)	96 (76)	132 (79)	
Irregular	65 (22)	30 (24)	35 (21)	
**Emergency attendance**				0.70
Attended	171 (58)	108 (86)	63 (38)	
Never attended	103 (42)	18 (14)	85 (51)	
**Admission**				0.54
Ever admitted	162 (55)	90 (71)	72 (43)	
Never admitted	131 (45)	36 (29)	95 (57)	
**Reason for last emergency visit**				0.56
Relapse	111 (38)	54 (43)	57 (34)	
Overdose	13 (5)	10 (8)	3 (2)	
Run out of medications	79 (27)	36 (29)	43 (26)	
Suicidal attempts	30 (10)	16 (13)	14 (8)	
Others	60 (20)	24 (19)	36 (22)	
**Reasons for last admission**				0.92
Relapse	138 (47)	64 (51)	74 (44)	
Overdose	22 (7)	10 (8)	12 (7)	
Severe depression	38 (13)	22 (17)	16 (10)	
Suicidal attempts	22 (7)	12 (9)	10 (6)	
Others	73 (25)	36 (29)	37 (22)	
**Health service barriers**				
Other thinking	94 (32)	44 (35)	50 (30)	0.36
Treatment failure	87 (30)	40 (32)	47 (28)	0.50
Confidentiality	79 (27)	39 (31)	40 (24)	0.06
Appointment availability	68 (23)	32 (25)	36 (22)	0.21
Family reaction	67 (23)	34 (27)	33 (20)	0.14
Colleague trust	66 (23)	35 (28)	31 (19)	0.05
Lack of information about services	62 (21)	30 (24)	32 (19)	0.33
Service accessibility	60 (20)	26 (21)	34 (20)	0.95
Appointment waiting time	53 (18)	23 (18)	30 (18)	0.83
Lack of personal time	41 (14)	30 (24)	11 (7)	0.03
**Perception of outcome of service**				0.62
Improved	199 (68)	87 (69)	112 (67)	
No improvement	94 (32)	39 (31)	55 (33)	

Patients in treatment for cessation (or reduction) of psychoactive substance use reported a wide range of hurdles to seeking health services. These ranged from social stigma to lack of time. Overall, a majority (68%) reported improvement in their condition upon attending healthcare services, while 32% did not perceive any improvement. The data showed us that the reported indices of utilization of healthcare services were comparable in Younger and Older groups.

### Factors associated with the perception of “improvement”

Table 4 indicates evaluative results for the associations between selected socio-demographic data, clinical indices, and perception of improvement upon receiving healthcare services. This was obtained using multivariate logistic regression modeling. Perception of improvement was significantly associated with being in the Younger group, having obtained pre-secondary education, and using a single psychoactive substance. Among the range of substances used, reported improvement was significantly associated with participants that used either cannabis or benzodiazepines.

**Table 4:** Factors associated with”improvement” perception upon receiving healthcare among attendees seeking consultation for alcohol and substance use at Al Masarra Hospital, Oman, from June 2013 to June 2014.

Characteristic	Improved	Not improved	OR	95% CI	P value
Younger age (<30 y)	81 (64)	45 (36)	1.92	(1.01, 3.57)	0.05
Muscat Region	160 (65)	70 (36)	1.05	(0.68, 1.61)	0.82
Currently married*	69 (68)	33 (32)	3.24	(0.16, 66.7)	0.74
Pre-secondary education	97 (63)	56 (37)	1.98	(1.18, 3.31)	0.01
Being unemployed	109 (71)	44 (29)	1.03	(0.79, 1.30)	0.97
Age of onset (<20 years)	132 (69)	60 (31)	1.11	(0.68, 1.19)	0.49
Non-injection intake	58 (59)	40 (41)	1.05	(0.83, 1.32)	0.67
Single substance use	18 (60)	12 (40)	1.55	(1.04, 2.32)	0.03
**Types of** substances **used**					
Opioids	158 (70)	69 (30)	1.39	(0.78, 2.47)	0.25
Cannabis	148 (72)	59 (29)	1.72	(1.02, 2.91)	0.04
Alcohol	128 (69)	57 (31)	1.17	(0.71, 1.94)	0.54
Heroin	117 (69)	53 (31)	1.10	(0.67, 1.81)	0.69
Benzodiazepines	112 (76)	36 (24)	2.07	(1.25, 3.42)	0.004
Cocaine	24 (39)	37 (61)	0.67	(0.37, 1.19)	0.17
Inhalants	17 (77)	5 (28)	1.05	(0.83, 1.32)	0.67

* The index category in marital status was “currently being married” versus the reference category which was a collapsed category of “currently unmarried” that included: being single, divorced, or widow.

## Discussion

The main goal of this study was to expand the literature surrounding substance use in Oman and those who seek treatment as there is little information that currently exists about this population. This study explored socio-demographic factors, factors related to the initiation of substance use, types of substances used, comorbid health and psychiatric conditions, healthcare utilization and hurdles to accessing healthcare among people that use psychoactive substances in a substance use disorder treatment facility in Oman. Other than certain impressionistic observations [2, 6, 28], no specific study on the aforementioned variables has been conducted in Oman before.

In terms of socio-demographic background, this study supports the view that consumption of alcohol and illicit substance use occurs predominantly among males. In previous studies from the US, fewer females than males were reported as struggling with issues relating to substance use [42, 43]. More recently, the narrowing gender gap has also caused the gap in male-female substance use to taper. This trend exists, however, only in ‘wet’ countries, i.e. those countries with relaxed policies toward certain psychotropic substances (such as cannabis) as well as alcohol [44, 45]. It also appears in relatively ‘dry’ countries like Oman, and others that are in transition such as those in Asia, Africa and South America [46–49]. There were only three Omani females that reported seeking therapy for cessation of psychoactive substance use in our study. This observed gender gap in alcohol and illicit substance use in Oman may partly stem from the socio-cultural circumstances of Oman being a patriarchal society that practices strict gender segregation. This. in turn, causes culturally disapproved behaviors among women to be practiced furtively [50].

Regarding the age of onset, most subjects indicated that they first experimented with psychoactive substances between ages 15 and 19. Approximately 16% admitted to starting even younger: between 10 and 14 years. This resonates with the international trend of exposure to controlled substances occurring typically during primary and secondary years of schooling [40, 51, 52]. This study also found that the percentage of substance use was higher among those whose education was limited to secondary or pre-secondary school levels, than among patients that had pursued higher education. In a study conducted in Saudi Arabia, Sheikh et al. [53] examined the quality of life among people that engage in substance use and those that do not. The study found that substance use was associated with lower educational and socio-economic status. This would imply that extending better educational opportunities for everyone might be a possible way of reducing the potential for substance use disorders. On the other hand, early initiation to psychoactive substances might negatively affect the student’s motivation to study further. Future studies should examine the interplay of the aforementioned factors and, additionally, the complex effects of individual differences—such as academic aptitude, intelligence, emotionality, and neurological vulnerability to substance use.

Studies from USA [54], Kuwait [55] and Saudi Arabia [56] have all shown that the availability and type of psychoactive substances, the individual’s personality, and peer pressure strongly influence the initiation of alcohol and substance use. Most of these factors were not explored in this study. A previous study conducted in Oman among a nationally representative school sample concluded that psychoactive substance initiation often occurs in the context of the influence of peers [21]. The present study also indicates a similar trend.

With regard to alcohol, a previous study in Oman [2] has suggested that alcohol use is strongly related to accessibility. According to McCabe et al. [57], the term “polysubstance use” refers to “the use of more than one psychoactive substance during a given period (e.g., 12 months) but not necessarily at the same time” (p 530). In American studies, the 12-month prevalence for polysubstance use was estimated to vary widely from 12.1% [57] to 64.4% [58] and 72.6% [59]. In our study, most patients engaged in polysubstance use.

Intravenous psychoactive substance use has been reported in many parts of the world and appears in different contexts within the Arabian Peninsula as well [6, 60–63]. Data from the World Health Organization, as reported by Panduranga, Al-Abri & Al-Lawati [61], mentions the prevalence of substance use in the Eastern Mediterranean region (which include Oman) to be 3,500/100,000 while prevalence of intravenous substance use disorders is at 172/100,000 people. According to the 2017 report of the United Nations Office on Drugs and Crime, the prevalence of injecting psychoactive substances has been estimated at 0.2% of the population in the region defined as “Near and the Middle East” (which includes Oman) [64]. In the present study, approximately 67% of the participants admitted having used intravenous substances.

The most consumed substances were opioids (including heroin), cannabis and alcohol. As often the case, accessibility defines the type of controlled substances that are more likely to be used. Oman lies close to the opioid and cannabis producing “Golden Crescent” region of Central Asia. Alcohol availability has increased with the influx of expatriate contract workers from different parts of the world who currently constitute about 45% of the total population, majority of whom are young, single men [30, 65]. The growth of the tourism industry has also contributed to easier availability of alcohol [15]. This observation is supported by existing literature [66, 67]. Not surprisingly, availability and accessibility appeared to be major factors that facilitated substance use initiation and dependence among the participants of this study as well.

Substance use has been known to compromise several health parameters, which are likely to worsen if administered intravenously [68, 69]. With reference to the data in 2007 from the WHO’s Oman *Atlas of Substance Use Disorders* [20], among 384 Omanis that were documented to inject themselves with psychoactive substances, 6.5% had HIV/AIDS, 42.5% had contracted Hepatitis B and 3.9% had Hepatitis C. In our study, Hepatitis C infection was the most common (41.3%) disease, followed by HIV/AIDS (4.1%), Hepatitis B virus (3.1%) and Tuberculosis (0.3%). The trend in Oman appears to echo the situation in regions of Bahrain and Saudi Arabia [62, 63]. If the link between people who inject psychoactive substances and proliferation of the infectious diseases would withstand further scrutiny, Oman ought to contemplate evidence-based prevention [70, 71].

Various studies from the Arabian Gulf have reported a strong link between the presence of psychiatric disorders and personality disorders and the development of substance use disorders [6, 72, 73]. Similar trends have emerged among other populations where the data has unequivocally suggested a higher incidence of psychiatric disorders among people with substance use disorders compared to the general population [74, 75, 76]. What is in question, however, is the direction of causation. One assumption is that substance use is a form of “self-medication for existing mental disorders” [77]. Another assumption is that consuming harmful substances might trigger mental disorders among a vulnerable population [78]. In the present study, 35% of the patients exhibited depressive symptoms while 18% had made suicidal attempts. This view is consonant with the literature suggesting a strong link between depressive symptoms/suicidality and substance use [79–82].

It has been previously documented that individuals in the Arabian Peninsula tend to hide substance use and associated problems partly due to perceived legal consequences and the strong social stigma in Islamic communities, where the consumption/use of any form of alcohol or psychoactive substances is perceived as *haram* (behavior forbidden by God) [83,84]. Given such background, traditional healing practices and illegal use of prescriptive medicines are rife and remain as the only recourse for people with substance use disorders in Arab countries [6, 85, 86]. Thus, people with substance use disorders in the Arabian Peninsula have been reported as receiving suboptimal rehabilitation, often resulting in relapse and fatalities [56, 87, 88].

In order to lay the groundwork for mitigating such trends, this study sought to identify the pattern of healthcare utilization that included gauging the number of and reasons for outpatient and emergency visits and admissions. The perceived barriers to and outcomes of services were also sought. Indeed, most patients admitted for substance use disorders in Oman appear to do so via emergency units. Relapses are common and associated with re-admissions, severe depression, overdose, and suicide attempts.

Oman, like most countries in the Arab world, represents a collectivistic community where individuals are socialized to suspend ‘selfhood’ for collective wellbeing [50]. Anthropological studies have described such societies as making strong use of honor and shame to enforce socialization and, as a result, individuals are accustomed to being preoccupied with what others think [89]. However, it is important to note here that it may not just be honor and shame being enforced during socialization, but also a promotion of community values, collective well-being, a feeling of belongingness and perceived social support which are all likely to have positive impacts on long-term individual well-being [90]. Nevertheless, while the majority of patients endorsed that the interventions at the rehabilitation center had improved their wellbeing, they were apprehensive of returning to their communities which might now perceive them as an outsider. This prospect appeared to dampen their motivation to attend therapy for cessation.

As with any study, this study also presents its own set of limitations. First, the present sample population of those attending a tertiary care facility might have been self-selective and a convenience sample. Hence, the generalizability is limited. More representative data is likely to emerge from a community study. This is particularly significant for a collectivistic and traditional society like Oman where substance and alcohol use remain furtive activities and rehabilitation is often in response to police/judicial demands or when an individual’s condition has reached advanced stages of pathology. This may explain the preponderance of polysubstance and intravenous substance use within our sample. It is likely that the individuals engaging in substance use and experiencing less acute problems tend not to seek biomedical help. Another limitation is the location of the healthcare unit. Oman has a total land area of 309,500 square kilometers with its population dispersed in different corners of the country separated by vast stretches of desert land. This study is limited to attendees seeking help from a tertiary care center located in the most urbanized northern coastal region of the country. The issue of urban-rural dichotomy was not considered in this study. The length of hospitalization was also not assessed. Studies have indicated a strong positive relationship between the length of hospitalization and good prognostic indicators [91]. Another potential limitation in the analysis is related to stratifying the database by two dichotomous categories of age and not analyzing age as a continuous variable. This dichotomous division simplified the presentation of data and facilitated conducting binary categorical data analysis. As reported by Altman & Royston [41, 92], this approach however, might have introduced the drawbacks of reduction in statistical power to detect a relation between age as a continuous variable and indices of substance use and underestimation of the extent of variation in substance use between Younger and Older groups. There is also the issue of concealment of any non-linearity in the relationship between age as a continuous variable and indices of substance use which might have affected the underlying assumptions of logistic regression modeling done as part of this study. Finally, the study is based on subjective self-reports which are known to be prone to social desirability bias.

## Conclusion

The current study is one of the first of its kind in Oman: a study shedding light on sociodemographic variables, factors influencing initiation to substance use, types of substance use, co-morbidity conditions, healthcare utilization and hurdles to access the same among patients with substance use disorder attending a tertiary substance use disorder rehabilitation unit. The present data demonstrates that young males in treatment for cessation of psychoactive substance use, especially those in their late teens, are at greater risk for alcohol and substance use, and peer pressure significantly contributes to the same. There is a significant association between intravenous substance use, the number of admissions, and testing positive for Hepatitis C. The sample population that was categorized as being younger than 30 years old, with primary education, and using a single substance such as cannabis and benzodiazepines, tended to have positive prognostic indicators. It is imperative that healthcare policy makers improve prevention strategies. Special emphasis must be placed on schools when discussing avoidance of associated co-morbidities. Efforts should be made to introduce effective measures in order to reduce expensive hospitalization and emergency visits.

## Supporting information

S1 Study questionnaire English. (DOCX)

S2 Study questionnaire Arabic. (DOCX)
