# Supplementary material for: The characteristics and patterns of utilization of healthcare services among Omanis with substance use disorders attending therapy for cessation
Source: PLoS One. 2019 Jan 31;14(1):e0210532. doi: 10.1371/journal.pone.0210532 (PMC6354979; doi:10.1371/journal.pone.0210532)
Supplement: S1 Study questionnaire — (DOCX) [file pone.0210532.s001.docx]

**STUDY QUESTIONNAIRE - English**

**The characteristics and patterns of utilization of healthcare services among Omanis with substance use disorders attending therapy for cessation**

1. **Socio-demographic data:**

**Q1. Age**: _____ years **Q2. Gender**: Male  Female

**Q3. Address**

| 1 | Muscat |  | 2 | Batinah North |  | 3 | Batinah South |  | 4 | Dakhilya |  |
| --- | --- | --- | --- | --- | --- | --- | --- | --- | --- | --- | --- |
| 5 | Shargiya North |  | 6 | Shargiya South |  | 7 | Dhahira |  | 8 | Buraimi |  |
| 9 | Al Wasta |  | 10 | Musandam |  | 11 | Dhofar |  |  | | |

**Q4. Current Marital Status**

| 1 | Single |  | 2 | Married |  | 3 | Divorced |  | 4 | Widow |  |
| --- | --- | --- | --- | --- | --- | --- | --- | --- | --- | --- | --- |

**Q5. Level of education**

| 1 | Illiterate |  | 2 | Read and write |  | 3 | Elementary school |  | 4 | Preparatory school |  |
| --- | --- | --- | --- | --- | --- | --- | --- | --- | --- | --- | --- |
| 5 | Secondary school |  | 6 | University/College |  | 7 | Postgraduate |  |  | | |

**Q6. Current employment status**

1 Unemployed

2 Employed

(a) Government  (b) Private  (c) Housewife  (d) Retired

3 Student

**Q7. Where do you live?**

1 In your house

2 In your parents’ house

3 In a rented house

4 Other

**Psychoactive drug history:**

| **Drug Category**  Which Drugs Ever Used | Ever used  1= Yes;  2 = No | | Total years  used | Injection Drug Use  1= Yes; 2 = No  3 = Not applicable | | | Year last used |
| --- | --- | --- | --- | --- | --- | --- | --- |
|  | 1 | 2 |  | 1 | 2 | 3 |  |
| ALCOHOL |  |  |  |  |  |  |  |
| CANNABIS:  Marijuana, hashish, hash oil |  |  |  |  |  |  |  |
| STIMULANTS  Cocaine, crack |  |  |  |  |  |  |  |
| BENZODIAZEPINES/TRANQUILIZERS:  Diazepam |  |  |  |  |  |  |  |
| HEROIN |  |  |  |  |  |  |  |
| OTHER OPIOIDS:  Morphine |  |  |  |  |  |  |  |
| HALLUCINOGENS:  LSD, PCP,STP,MDA,DAT, mescaline, peyote, mushrooms, ecstasy (MDMA) Nitrous Oxide |  |  |  |  |  |  |  |
| INHALANTS:  Glue, gasoline, aerosols, paint thinner, poppers, rush, locker room |  |  |  |  |  |  |  |
| OTHER:  (Specify) |  |  |  |  |  |  |  |

1. **Risk factors for substances abuse: why**

Q1 At what age did you start your first substance abuse? _____ years

Q2 What made you to start your first attempt?

1. Out of curiosity
2. To have a good time
3. Because friends are doing it
4. To ease another problem, such as stress, anxiety, or depression
5. Other

Q3 Is alcohol easy to get? Yes  No

Q4 Are the psychoactive drugs easy to get Yes  No

Did you have any traumatic experiences in childhood? Yes  No  **If yes, specify**

1. Physical abuse
2. Sexual abuse
3. Neglect
4. Other

Q5 Is there any family members who are addict to any substance Yes  No

If yes, Who?

1. **Comorbidities**

Q1 Do you have any medical problem?

Does/Did it limit your activity Yes  No

| Problem | | Do/Did you have the problem (Yes/No) | | Do/Did you receive treatment (Yes/No) | | Does/Did it limit your activity (Yes/No) | |
| --- | --- | --- | --- | --- | --- | --- | --- |
|  |  | 1 Yes | 2 No | 1 Yes | 2 No | 1 Yes | 2 No |
| a | HIV |  |  |  |  |  |  |
| b | HCV |  |  |  |  |  |  |
| c | HBV |  |  |  |  |  |  |
| d | TB |  |  |  |  |  |  |
| e | Depression |  |  |  |  |  |  |
| f | Suicidal attempt |  |  |  |  |  |  |
| g | Urethral/Vaginal discharge |  |  |  |  |  |  |
| h | Any other (Specify) |  |  |  |  |  |  |
|  |  |  |  |  |  |  |  |

1. **Health care utilization**

| 1. **Outpatient Hospital Care ( last 4 years)** | | |
| --- | --- | --- |
| 1. Are you attending your OPD Clinic appointments regularly (1 yes; 2 No) | Yes  No | |
| 1. When was your first visit to Al Massara Hospital | < = 1 years ago  2 years ago | 3 years ago  ≥4 years ago |
| 1. How many appointments did you attend for the last 4 years? |  | |
| 1. How frequently you get appointments | 1. Monthly 2. 2. Every 3 month 3. Every 6 month 4. Yearly | |
| 1. Who fixed for you the appointment? | 1. Police 2. Family 3. Self 4. Friends 5. Others | |

| 1. **Emergency Care (4 last 4 years)** | |
| --- | --- |
| 1. For the last 4 years how many times have you attended Al Massara Hospital Emergency Clinic |  |
| 1. When was your last emergency visit to Al Massara Hospital? |  |
| 1. What was the reason for your last emergency visit? | 1. Relapse 2. Overdose 3. Run out of medicine 4. Suicidal attempt 5. Other |
| 1. Did you get admitted during your last emergency visit? (1Yes /2 No ) | 1. Yes 2. No |

| 1. **Inpatient Hospital Care (4 last 4 years)** | |
| --- | --- |
| 1. In the last 4 years, have you ever been admitted in Al Massara Hospital? | 1= Yes  2= No |
| 1. When was your last emergency visit to Al Massara Hospital? |  |
| 1. Which reason best describes why you were admitted last hospitalized? | 1. Relapse 2. Overdose 3. Severe depression 4. Suicidal attempt 5. Other |
| 1. How did you get there? | 1. Private vehicle 2. Public transportation 3. Taxi 4. Ambulance 5. Walked 6. Do not know |
| 1. Thinking about your last (hospital) stay, did any of your family members visit you? | 1. Yes 2. No |
| 1. Overall, how satisfied were you with the care you received during your last admission? | 1. Very satisfied 2. Satisfied 3. Neither satisfied nor dissatisfied 4. Dissatisfied 5. Very dissatisfied |
| 1. What was the outcome or result of your visit to the (hospital)? Did your condition …. | 1. Get much better 2. Get better 3. No change 4. Get worse 5. Get much worse |

1. **Barriers of Health Care Utilization**

Q1. **Why you didn’t seek health care advice for your substance abuse problem?**

1. You are concern about confidentiality
2. Yu are concern about what others think
3. Your colleague will have less confidence in you
4. You don’t believe the treatment would help you
5. Your family will fire you out from home
6. Long waiting lists for appointment availability
7. Long appointment waiting time
8. Time (lack of personal time to seek help)
9. Lack of convenient access to care location
10. Lack of information about how and where to obtain services

Thank you
