# Supplementary material for: The characteristics and patterns of utilization of healthcare services among Omanis with substance use disorders attending therapy for cessation
Source: PLoS One. 2019 Jan 31;14(1):e0210532. doi: 10.1371/journal.pone.0210532 (PMC6354979; doi:10.1371/journal.pone.0210532)
Supplement: S2 Study questionnaire — (DOCX) [file pone.0210532.s002.docx]

**STUDY QUESTIONNAIRE - Arabic**

1. **بيانات حول الحالة الاجتماعية السكانية**

|  | سنة--------------- | | العمر | س1 |
| --- | --- | --- | --- | --- |
|  | 1. ذكر | | الجنس | س2 |
|  | 1. انثى | |  |  |
|  | 1. مسقط | | العنوان | س3 |
|  | 1. شمال الباطنة | |  |  |
|  | 1. جنوب الباطنة | |  |  |
|  | 1. الداخلية | |  |  |
|  | 1. شمال الشرقية | |  |  |
|  | 1. جنوب الشرقية | |  |  |
|  | 1. الظاهرة | |  |  |
|  | 1. البريمي | |  |  |
|  | 1. الوسطى | |  |  |
|  | 1. مسندم | |  |  |
|  | 1. ظفار | |  |  |
|  | 1. اعزب | | الحالة الزوجية | س4 |
|  | 1. متزوج | |  |  |
|  | 1. مطلق | |  |  |
|  | 1. أرمل | |  |  |
|  | 1. امي | | مستوى التعليم | س5 |
|  | 1. يقرا ويكتب | |  |  |
|  | 1. ابتدائي | |  |  |
|  | 1. اعدادي | |  |  |
|  | 1. ثانوي | |  |  |
|  | 1. جامعي | |  |  |
|  | 1. اكثر | |  |  |
|  | 1. عاطل عن العمل | | الوظيفة | س6 |
|  |  | 1. موظف في : |  |  |
|  | 1. قطاع حكومي |  |  |  |
|  | 1. قطاع خاص |  |  |  |
|  | 1. ربة بيت | |  |  |
|  | 1. متقاعد | |  |  |
|  | 1. طالب | |  |  |
|  | 1. ملك خاص | | مكان المعيشة | س7 |
|  | 1. بيت الاسرة | |  |  |
|  | 1. بيت بالايجار | |  |  |
|  | 1. أخرى | |  |  |

ب. تاريخ العقاقير المنشطه

| د.اخر سنه تم التعاطي فيها؟  20----------- | ج.التعاطي بالابر ؟   1. لا 2. نعم 3. لا ينطبق | ب. عدد سنوات الاستخدام؟ | ا.هل استخدمت العقاقير التالية سابقا/حاليا ؟  1=لا  2=نعم | تصنيف العقار | س |
| --- | --- | --- | --- | --- | --- |
|  |  |  |  | الكحول | 1 |
|  |  |  |  | مجموعة الحشيش:  المرجوانا/ الحشيش | 2 |
|  |  |  |  | المحفزات:  الكوكايين | 3 |
|  |  |  |  | المهدئات:  الديازيبام | 5 |
|  |  |  |  | الهيروين | 6 |
|  |  |  |  | المواد الافيونية :  المورفين | 7 |
|  |  |  |  | المستنشقات:  الغرا/ الجازولين/مرقق الدهان/الرش | 8 |
|  |  |  |  | اخرى:  حدد---------------- | 9 |

السؤال ( ج) : العوامل المؤدية للإدمان :

|  | سنة | | بأي عمر بدأت بتعاطي أول جرعة مخدر أو مهلوس ؟ | س1 |
| --- | --- | --- | --- | --- |
|  | 1. بدافع الفضول | | ما أسباب بدأك سلوك طريق الادمان ؟  ما الدي جعلك تبدا اول محاولة ؟ | س2 |
|  | 1. للاستمتاع | |  |  |
|  | 1. لأن أصحابي يتعاطون أيضاً | |  |  |
|  | 1. للتخفيف عن بعض المشاكل (كالتوتر والاكتئاب والقلق) | |  |  |
|  | 1. أسباب أخرى ( يرجى ذكرها ): | |  |  |
|  | 1. نعم | | هل من السهل الحصول على الكحوليات ؟ | س3 |
|  | 1. لا | |  |  |
|  | 1. نعم | | هل من السهل الحصول على المخدرات؟ | س4 |
|  | 1. لا | |  |  |
|  | 1. اعتداء جسدي | 1. نعم   ( ماهي ؟ ) | هل لديك أي تجارب مؤلمة في مرحلة الطفولة؟ | س5 |
|  | 1. اعتداء جنسي |  |  |  |
|  | 1. الاهمال |  |  |  |
|  | 1. غيرها |  |  |  |
|  | 1. لا | |  |  |
|  | 1. نعم  - ما هي صلة القرابة ؟ ادكرها | | هل هناك أحد آخر من أهلك وأقاربك من هو \ هي متعاطي ومدمن ؟ | س6 |
|  | 1. لا | |  |  |

د.الحالات المرضية المصاحبة

|  | هل لديك أي مشكلة صحية؟ | | | | | | | | س |
| --- | --- | --- | --- | --- | --- | --- | --- | --- | --- |
|  | هل المشكلة تحد من النشاط الخاص بك؟ | | هل تلقيت العلاج لذلك؟ | | هل لديك مشكلة صحية؟ | | المشكلة الصحية | 1 | |
|  | نعم  1 | لا  2 | نعم  1 | لا  2 | نعم  1 | لا  2 |  |  |  |
|  |  | |  | |  | | فيروس نقص المناعة البشرية  (HIV) | 2 | |
|  |  | |  | |  | | الوباء الكبدي ج  (HCV) | 3 | |
|  |  | |  | |  | | الوباء الكبدي ب  (HBV) | 4 | |
|  |  | |  | |  | | مرض السل  (TB) | 5 | |
|  |  | |  | |  | | الاكتئاب  (Depression) | 6 | |
|  |  | |  | |  | | محاولة انتحار  (Suicidal attempt) | 7 | |
|  |  | |  | |  | | إحليلية / افرازات مهبلية (Urethrral/vaginal discharge) | 8 | |
|  |  | |  | |  | | أخرى (حدد) | 9 | |

**هـ. الاستفادة من الخدمات الصحية**

| **س 1. خدمات العيادة الخارجية (السنوات الأربع الأخيرة)** | | | | | | |
| --- | --- | --- | --- | --- | --- | --- |
|  | 1. نعم | | | هل تحرص على حضور مواعيد العيادة الخارجية بانتظام ؟ | | 1.1 |
|  | 1. لا | | |  |  |  |
|  | 1. منذ أقل من سنه | | | متى كانت آخر زيارة لك لمستشفى إبن سينا؟ | | 1.2 |
|  | 1. منذ سنتين | | |  |  |  |
|  | 1. منذ ثلاث سنوات | | |  |  |  |
|  | 1. منذ اربع سنوات او اكثر | | |  |  |  |
|  | ..................موعد | | | كم عدد المواعيد التي حضرتها في آخر أربع سنوات؟ | | 1.3 |
|  | 1. شهريا | | | ما هي الفترة الزمنية الفاصلة بين مواعيدك؟ | | 1.4 |
|  | 1. كل ثلاثة أشهر | | |  |  |  |
|  | 1. كل ستة أشهر | | |  |  |  |
|  | 1. سنويا | | |  |  |  |
| **س 2 . زيارت غرفة الطوارئ (السنوات الأربع الأخيرة)** | | | | | | |
|  | | ( ) مرة | | كم مرة اضطررت للحضور إلى قسم طورائ مستشفى ابن سينا في آخر أربع سنوات؟ | | 2.1 |
|  | | ................... | | متى كانت آخر زيارة لك لقسم الطوارئ؟ | | 2.2 |
|  | | 1. انتكاسة | | ما هو سبب زيارتك الأخيرة لقسم الطوارئ | | 2.3 |
|  | | 1. جرعة زائده | |  |  |  |
|  | | 1. نفاد الأدوية التي تستخدمها | |  |  |  |
|  | | 1. محاولة انتحار | |  |  |  |
|  | | 1. أخرى | |  |  |  |
|  | | 1. نعم 2. لا | | هل تم ترقيدك في المستشفى في آخر زيارة لك لقسم الطوارئ؟ | | 2.4 |
| **س 3 .خدمات رعاية المرضى الداخلية أو الترقيد (للسنوات الأربع الأخيرة)** | | | | | | |
| 1 | | 1. نعم | هل سبق وأن تم ترقيدك بمستشفى ابن سينا خلال الاربع سنوات الماضية؟ | | 3.1 | |
|  |  | 2. لا |  |  |  |  |
|  | | 1.قبل عام | متى كانت آخر مرة تم ترقيدك فيها بمستشفى ابن سينا | | 3.2 | |
|  | | 2. قيل شهر |  |  |  |  |
|  | | 3. قبل اسبوع |  |  |  |  |
|  | | قبل يوم |  |  |  |  |
|  | | 1. انتكاسه | ما هو الخيار الأنسب الذي يبين سبب ترقيدك آخر مرة من بين الخيارات التاليه ؟ | | 3.4 | |
|  |  | 2. جرعه زائده |  |  |  |  |
|  |  | 3 . اكتئاب شديد |  |  |  |  |
|  |  | 4 . محاولة انتحار |  |  |  |  |
|  |  | 5. أخرى |  |  |  |  |
|  | | 1. سياره خاصه | كيف وصلت إلى المستشفى يومئذ؟ | | 3.5 | |
|  |  | 2. وسيلة نقل عامه |  |  |  |  |
|  |  | 3. سيارة أجره |  |  |  |  |
|  |  | 4. سيارة اسعاف |  |  |  |  |
|  |  | 5. مشيا على الاقدام |  |  |  |  |
|  |  | 6. لا أدري |  |  |  |  |
|  | | 1. نعم | بالرجوع إلى آخر إقامة لك بالمستشفى , هل قام بزيارتك خلال إقامتك أي من أفراد أسرتك؟ | | 3.6 | |
|  |  | 2. لا |  |  |  |  |
|  | | 1. راضي تماما | إجمالا, ما مدى رضاك عن الرعاية الصحية التي تلقيتها خلال إقامتك الأخيرة بالمستشفى؟ | | 3.7 | |
|  |  | 2. راضي |  |  |  |  |
|  |  | 3. محايد او متعادل (لا أشعر بالرضا او عدم الرضا) |  |  |  |  |
|  |  | 4. غير راضي |  |  |  |  |
|  |  | 5. غير راضي أبدا |  |  |  |  |
|  | | 1. تحسنت بشكل كبير | كيف كانت نتيجة آخر زيارة لك للمستشفى ؟ هل حالتك .. | | 3.8 | |
|  |  | 2. تحسنت |  |  |  |  |
|  |  | 3. لن تتغير |  |  |  |  |
|  |  | 4. ساءت |  |  |  |  |
|  |  | 5. ساءت كثيرا |  |  |  |  |

**ف. حواجز استخدام الرعاية الصحية**

| س1. لماذا لم تسعى للمشورة الصحية الخاصة بمشكلة تعاطي المخدرات؟  ضع علامة صح على الإجابات المناسبة)) | | | |
| --- | --- | --- | --- |
|  |  | كنت قلقا حول السرية ف المعلومات. | ا |
|  |  | انت قلق على ما يعتقده الاخرون عنك | ب |
|  |  | سوف تقل ثقة اصدقائك بك | ج |
|  |  | كنت لا تصدق ان العلاج سوف يفيدك | د |
|  |  | كنت تعتقد ان اهلك سوف يطردوك من المنزل | ه |
|  |  | قوائم انتظار المواعيد طويله | و |
|  |  | الانتظار الطويل قبل الدخول عند الطبيب في الموعد المحدد | ز |
|  |  | ضيق الوقت الشخصي لطلب المساعدة | ح |
|  |  | صعوبة الوصول الى مكان العلاج او الرعاية | ط |
|  |  | عدم وجود معلومات حول كيفية الحصول على الخدمات ومكانها | ي |
